# Photodynamic Therapy Combined with Ferroptosis Is a Synergistic Antitumor Therapy Strategy

**DOI:** 10.3390/cancers15205043

**Published:** 2023-10-19

**Authors:** Yunpeng Huang, Xiaoyu Li, Zijian Zhang, Li Xiong, Yongxiang Wang, Yu Wen

**Affiliations:** 1 Department of General Surgery, The Second Xiangya Hospital of Central South University, Changsha 410011, China; 218202108@csu.edu.cn (Y.H.); 178211082@csu.edu.cn (Z.Z.); lixionghn@csu.edu.cn (L.X.); 2 Department of Obstetrics and Gynecology, The Second Xiangya Hospital of Central South University, Changsha 410011, China; 188211103@csu.edu.cn

**Keywords:** photodynamic therapy, ferroptosis, nanoparticles, synergistic therapy, antitumor therapy

## Abstract

**Simple Summary:**

In this work, we focused on the synergistic antitumor effects of photodynamic therapy and ferroptosis. First, we briefly introduced the basic theory of ferroptosis and photodynamic therapy. We explored the synergistic anti-tumor effect of photodynamic therapy combined with ferroptosis from a mechanism perspective. Secondly, we introduced the application of photodynamic therapy combined with ferroptosis, which mainly includes the construction of nanomaterials and drug combination. Nanomaterials can exert synergistic effects by activating anti-tumor immunity, improving the hypoxic microenvironment, and inhibiting tumor angiogenesis. The drug combination strategy has good application prospects and clinical significance.We also discussed the shortcomings of existing combination treatment strategies and potential solutions. In conclusion, photodynamic therapy combined with ferroptosis is a promising combination anticancer strategy.

**Abstract:**

Ferroptosis is a programmed death mode that regulates redox homeostasis in cells, and recent studies suggest that it is a promising mode of tumor cell death. Ferroptosis is regulated by iron metabolism, lipid metabolism, and intracellular reducing substances, which is the mechanism basis of its combination with photodynamic therapy (PDT). PDT generates reactive oxygen species (ROS) and ^1^O_2_ through type I and type II photochemical reactions, and subsequently induces ferroptosis through the Fenton reaction and the peroxidation of cell membrane lipids. PDT kills tumor cells by generating excessive cytotoxic ROS. Due to the limited laser depth and photosensitizer enrichment, the systemic treatment effect of PDT is not good. Combining PDT with ferroptosis can compensate for these shortcomings. Nanoparticles constructed by photosensitizers and ferroptosis agonists are widely used in the field of combination therapy, and their targeting and biological safety can be improved through modification. These nanoparticles not only directly kill tumor cells but also further exert the synergistic effect of PDT and ferroptosis by activating antitumor immunity, improving the hypoxia microenvironment, and inhibiting the tumor angiogenesis. Ferroptosis-agonist-induced chemotherapy and PDT-induced ablation also have good clinical application prospects. In this review, we summarize the current research progress on PDT and ferroptosis and how PDT and ferroptosis promote each other.

## 1. Introduction

### 1.1. Ferroptosis

**Figure 1:**
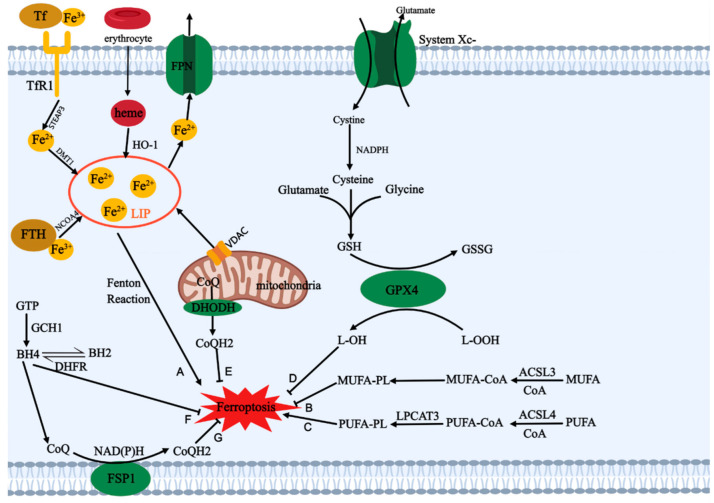
Regulatory mechanism of ferroptosis. (A) The iron metabolism pathway induces ferroptosis by Fenton reaction. (B, C) The effect of lipid metabolism on ferroptosis. PUFA promotes ferroptosis and MUFA inhibits ferroptosis. (D) Classic ferroptosis inhibition pathway. System Xc^−^ and GPX4 inhibit ferroptosis by promoting GSH and depleting lipid peroxides. (E–G) Non-traditional ferroptosis inhibition pathway.

Ferroptosis is a programmed cell death mode different from apoptosis, autophagy, pyroptosis, and necrosis, and the research on it has grown exponentially since it was first found in 2012 [1]. The occurrence of ferroptosis is mainly caused by the imbalance between intracellular oxidative damage and reductive protection, which is specifically represented in the depletion of GSH and intracellular overloading of free iron ions, ROS, and lipid peroxides (LPO) [2]. Ferroptosis primarily acts on the cellular mitochondria, leading to mitochondrial depolarization, increased membrane potential, decreased membrane density, rupture of the outer membrane, and decreased volume [3,4]. Ferroptosis leads to the death of normal cells (neuron cells, cardiomyocytes), which causes neurodegenerative disorders and ischemia-reperfusion injury [5,6]. For tumors, a variety of treatment methods (radiotherapy, chemotherapy, immunotherapy, and photodynamic therapy) can exert antitumor effects by inducing ferroptosis [7,8,9]. The traditional ferroptosis agonist erastin can cause ferroptosis, which can specifically target tumor cells with RAS mutations without harming healthy cells. Therefore, previous studies have shown that the RAS gene is a crucial component of ferroptosis in tumor cells [10,11]. However, with the further research in recent years, a large number of ferroptosis regulatory genes have been identified, which are classified as ferroptosis positive regulator genes (p53, TFR1, CARS, VDAC2/3, NOX) and ferroptosis negative regulator genes (SLC7A11, GPX4, HSPB1, NRF2) [12]. In addition, the regulatory mechanism of ferroptosis, including iron metabolism, lipid metabolism, and cellular antioxidant regulation pathways, has also been gradually improved (See Figure 1 for details).

Iron metabolism is one of the important ways to regulate ferroptosis. It regulates ferroptosis by changing the amount of Fe^2+^ in cells by controlling the labile iron pool (LIP). There are three main ways to increase the LIP in cells [13]: (1) Extracellular Fe^3+^ combined with transferrin is recognized by transferrin receptor 1 on the cell membrane surface and transported into the cell, where it is reduced to Fe^2+^ under the action of STEAP3, and, finally, transported to the LIP by DMT1. (2) The aging red blood cells in the blood circulation are decomposed into heme under the action of macrophages, and then catalyzed by heme oxygenase-1 (HO-1) to release Fe^2+^. (3) The ferritin heavy chain 1 (FTH1) in human ferritin can oxidize Fe^2+^ into Fe^3+^ and store it in the center of the protein. Fe^2+^ is released under the ferritinophagy mediated by nuclear receptor coactivator 4 (NCOA4). In addition, the LIP can also be increased by reducing the efflux of iron ions. Ferroportin 1 (FPN1) is a transporter that transports intracellular iron outward, and reducing the expression of FPN1 can also promote ferroptosis [14]. Free Fe^2+^ in cells can react with H_2_O_2_ through the Fenton reaction to generate hydroxide and hydroxyl radicals to induce ferroptosis. Overloaded iron ions can also promote lipid peroxidation and ferroptosis by activating iron-containing enzymes [15]. Interestingly, dietary iron overload can also induce ferroptosis by reducing the expression of GPX4 and SLC7A11 and mitochondrial membrane potential and promoting the expression of ACSL4 [16]. In addition, mitochondrial iron overload can also induce ferroptosis [17]. Iron ions in mitochondria can be used to synthesize iron–sulfur (Fe–S) clusters, and overexpression of the iron-sulfur cluster assembly enzyme can restore mitochondrial function and increase GSH levels to alleviate ferroptosis [18]. Similar to the cell matrix, mitochondrial ferritin (FtMt) is an iron storage protein in mitochondria, which can control the LIP in mitochondria and reduce ferritin in cytoplasm [19]. Studies have found that overexpression of FtMt can inhibit ferroptosis by regulating iron homeostasis [20]. Ablation of FtMt reduces ferroportin 1 and increases total and chelatable iron, thereby promoting ferroptosis [21]. In addition, iron ions in mitochondria can be transported through channels in the mitochondrial membrane. Iron ions can be transported through the voltage-dependent anion channel (VDAC) on the mitochondrial membrane, and the degradation of VDAC2/3 can inhibit ferroptosis [22]. Increased mitochondrial iron uptake by blocking the mitochondrial membrane proteins CDGSH iron sulfur domain 1 and CDGSH iron sulfur domain 2 can also induce ferroptosis [23,24].

Peroxidation of intracellular polyunsaturated fatty acids is another crucial way to induce ferroptosis. First, arachidonic acid (AA) and adrenal acid (Ada) are free fatty acids in the body, which can be activated by ASCL4 to link with CoA to produce AA/AdA CoA and form PUFA-PL under the esterification of LPCAT3 [25]. PUFA-PL can generate lipid peroxide through the peroxidation reaction in enzymatic and non-enzymatic ways, thereby destroying cell membrane integrity, impairing mitochondrial function, and inducing ferroptosis. The enzymatic way refers to the generation of lipid peroxides under the catalysis of ALOXs and POR. The Fenton reaction drives the non-enzymatic way, and Fe^2+^ reacts with PLOOH to generate lipid hydroxyl radicals (PLO•) and lipid peroxyl radicals (PLOO•). Inhibition of LPCAT3, ACSL4, ALOX15, and POR can reduce the degree of ferroptosis and protect cells [26,27,28,29]. However, monounsaturated fatty acid (MUFA) is a less oxidative fatty acid compared to PUFA, and MUFA-PL can replace polyunsaturated fatty acid in the cell membrane to inhibit lipid peroxidation and ferroptosis. Exogenous supplementation of MUFAs can inhibit ferroptosis under the activation of ACSL3 [30]. Oleic acid can also inhibit melanocyte ferroptosis and enhance metastasis in an ACSL3-dependent manner, and melanoma patients with high expression of ACSL3 have a lower survival rate [31]. Therefore, lipid metabolism can regulate and affect ferroptosis by regulating the balance between MUFA and PUFA in phospholipids.

Due to the existence of several reduced substances (GSH, COQH2, BH4) in cells, they can inhibit the occurrence of ferroptosis through redox reactions with ROS and lipid peroxides. Intracellular antioxidant regulation pathways are mainly divided into the classical pathway mediated by GPX4 (System Xc^–^/GSH/GPX4) and three non-traditional axes (FSP1/CoQ10/NAD(P)H, GCH1/BH4/DHFR, DHODH/CoQH2) [32]. System Xc^–^ is a cystine/glutamate membrane transport protein, which is divided into solute carrier family 7 member 11 (SLC7A11) and solute carrier family 3 member 2 (SLC3A2) [33,34]. It can transfer extracellular cystine into the cell while exchanging glutamate out of the cell. Intracellular cystine can consume NADPH and be oxidized to cysteine, and glutathione (GSH) is formed together with glutamic acid and glycine under the action of glutamic acid-cysteine ligase and glutathione synthetase [35]. Intracellular glutathione has two forms: reduced state (GSH) and oxidized state (GSSG), and the reciprocal transformation between them is an important process for maintaining cellular redox homeostasis [36]. Glutathione peroxidase 4 (GPX4) can oxidize GSH and reduce lipid peroxide to alcohol to inhibit ferroptosis, and it is the core protein in the classic ferroptosis inhibition pathway [37,38].

The first unconventional ferroptosis antagonistic pathway identified was the FSP1-CoQ10-NAD(P)H axis, which suppresses ferroptosis due to GPX4 deficiency [39,40]. Ferroptosis suppressor protein 1 (FSP1) is located on the cell membrane and lipid droplets, and after being modified by myristoylation, it can reduce COQ10 by consuming NAD(P)H to inhibit ferroptosis. In addition, the inhibition of COQ2 can lead to a decrease in the levels of COQ10 and an increase in sensitivity to ferroptosis, suggesting that ubiquinone may be crucial to the ferroptosis antagonistic system. Further studies found that dihydroorotate dehydrogenase (DHODH) prevents lipid peroxidation by reducing CoQ to CoQH2 in mitochondria [41]. DHODH, which is located in the inner mitochondrial membrane, regulates ferroptosis as well as pyrimidine nucleotide synthesis by catalyzing the oxidation of dihydroorotate to orotate. Knockdown of the expression of DHODH promotes ferroptosis in parallel with the effect of GPX4. GTP cyclohydrolase-1 (GCH1) is the rate-limiting enzyme for the synthesis of tetrahydrobiopterin (BH4). Inhibition of GCH1 can reduce the production of BH4 [42]. Studies have found that overexpression of GCH1 can promote the synthesis of BH4. BH4 can capture free radicals and inhibit ferroptosis through reducing lipid peroxidation [43,44]. Dihydrofolate reductase (DHFR) can catalyze the reduction of BH2 and regenerate BH4, and researchers have found that the oxidative degradation of DHFR can lead to increased sensitivity to ferroptosis in cells with a high expression level of CD38 [45,46]. BH4 can also regulate the de novo synthesis of COQ10 by promoting the conversion of phenylalanine to tyrosine and inhibit the occurrence of ferroptosis [44].

### 1.2. Photodynamic Therapy

**Figure 2:**
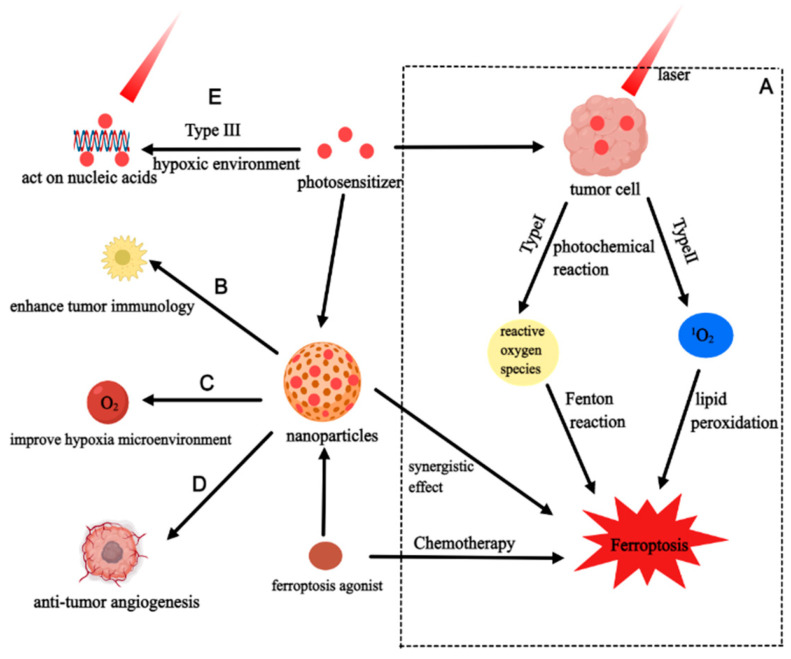
The mechanism of PDT and the synergistic effect of combined ferroptosis. (A) PDT can induce ferroptosis by photochemical reaction and exert a synergistic effect with ferroptosis agonists. (B–D) Nanoparticles coated with photosensitizers and ferroptosis agonists can exert a synergistic effect through enhancing tumor immunology, improving the hypoxia microenvironment and inhibiting tumor angiogenesis. (E) In a hypoxic environment, PDT can act on nucleic acids through a type III photochemical reaction.

Photodynamic therapy (PDT) is a rapidly emerging tumor therapy. Due to the enhanced permeation and retention effect of photosensitizers, PDT has certain tumor targeting properties [47]. Specifically, when a tumor is treated with PDT, photosensitizers generate singlet oxygen and reactive oxygen species through two kinds of photochemical reactions under the irradiation of laser light of specific wavelengths. A type I photochemical reaction means that the excited photosensitizer transfers electrons or protons to form cytotoxic reactive oxygen species (O_2_^−^•, H O_2_•, H O•, H_2_O_2_); a type II photochemical reaction means that the photosensitizer is excited into a triplet state (3PS•), and triplet–triplet interaction with molecular oxygen generates singlet oxygen (^1^O_2_) [48]. In fact, most tumors are in a hypoxic environment, and a type III photochemical reaction can kill tumor cells without ROS. A type III photochemical reaction means that some activated photosensitizers (such as Psoralens and Tetracyclines) can act on subcellular structures (proteins, nucleic acids, and other biological macromolecules), thereby producing a killing effect [49,50] (Figure 2).

With the development of endoscopy and light technology, PDT can be applied not only to surface tumors but also to solid tumors in vivo, including colorectal cancer [51], non-melanoma skin cancer [52], bladder cancer [53], liver cancer [54], cervical cancer [55], and breast cancer [56]. For non-solid tumors, PDT also has certain therapeutic effects. A randomized clinical trial confirmed that synthetic hypericin PDT can effectively relieve mycosis fungoides-cutaneous T cell lymphoma, and the index lesion response rate can reach 49% after three cycles of treatment [57]. Animal experiments have found that systemic PDT can effectively promote tumor cell apoptosis and inhibit the spread of tumors (leukemia and lymphoma) [58]. In addition, PDT is an effective treatment for some advanced unresectable tumors. For example, it can improve the progression-free survival of patients with recurrent inoperable glioblastoma [59], improve the survival rate of unresectable extrahepatic cholangiocarcinoma [60], and provide surgical opportunities for patients with stage III central non-small-cell lung cancer [61]. However, due to the short survival time of ROS (0.03–0.18 ms), limited laser penetration depth (<10 mm), and difficulty in covering the entire tumor, the efficacy of PDT alone in treating tumors is relatively limited [62]. Combining PDT with other treatment methods (chemotherapy, radiotherapy, surgery, and immunotherapy) can overcome the barriers to the application of PDT and can also make up for the shortcomings of other treatment methods. 

Previous studies have explored the possibility of combining PDT with chemotherapy, radiotherapy, immunotherapy, and surgery, and found that the combined treatment is superior to monotherapy. Combining PDT with chemotherapy can reduce the dose of chemotherapy drugs and improve the problem of multidrug resistance. Some clinical studies have also confirmed that PDT combined with chemotherapy is more effective in treating tumors than a single treatment. A 1992 study found that the complete remission rates of cardiac cancer patients who received PDT, chemotherapy, and combination therapy were 5.5%, 8.3%, and 19.5%, respectively [63]. Another clinical study confirmed that PDT combined with chemotherapy can significantly increase the survival time of patients with advanced cholangiocarcinoma compared with PDT alone (median survival time in the combination treatment group was 538 days, compared with 334 days in the PDT alone group) [64]. In addition, PDT combined with radiotherapy (high dose rate brachytherapy) can effectively locally control endobronchial non-small-cell lung cancer, and patients tolerated the combination treatment well [65]. The combination of PDT and surgery can eliminate the micro-residual tumor, which is beneficial to the prognosis of patients. A case report reported the treatment effect of combined PDT in lobectomy. The patient’s tumor was resected, and there were no signs of disease after 13 months of follow-up [66]. For non-small-cell lung cancer patients with pleural spread that cannot be controlled by surgery alone, PDT can enhance the therapeutic effect of surgery, and the median survival time of the combined treatment is 21.7 months [67]. PDT combined with immunotherapy is a complementary combination strategy. PDT can stimulate the immune response to kill colorectal cancer cells, and when combined with PD-L1 blockade can further enhance the immune response and inhibit tumor recurrence [68]. The study also found that PDT-induced hypoxia can promote the expression of PD-L1 in colorectal cancer, thereby sensitizing immunotherapy. Although the above combined strategies overcome the shortcomings of PDT, there are still some problems. First, although combined strategies can sensitize chemotherapy, radiotherapy, and immunotherapy, these treatments still have their own problems. For example, the systemic side effects of chemotherapy and immunotherapy, the damage of radiotherapy to surrounding tissues, and limited surgical indications. PDT kills tumors by generating ROS through O_2_, which will lead to hypoxia in the tumor microenvironment. The hypoxic microenvironment will lead to drug resistance to radiotherapy and chemotherapy, so improving the hypoxic microenvironment is an important direction for broadening the combination strategy [69,70]. 

Ferroptosis is a form of cell death that can be induced by multiple therapies, and the antitumor effect of it can be further enhanced by the reactive oxygen species generated by PDT. Combining ferroptosis with PDT could be a more comprehensive strategy.

### 1.3. Synergistic Effects of Photodynamic Therapy Combined with Ferroptosis

In terms of mechanisms, H_2_O_2_ produced by a type I photochemical reaction can induce ferroptosis with iron ions through the Fenton reaction, and ^1^O_2_ produced by a type II photochemical reaction can directly oxidize membrane lipids and promote lipid peroxidation. Therefore, Mishchenko proposed that PDT and ferroptosis had synergistic antitumor effects [71]. Next, we will discuss the underlying mechanism of their synergistic effects.

Recent studies have found that PDT can destroy tumor cells through a variety of unconventional cell death pathways, including: paraptosis, parthanatos, mitotic catastrophe, pyroptosis, necroptosis, and ferroptosis [48]. ROS and lipid peroxides required for ferroptosis are also the products of PDT in the process of eliminating tumors, and increasing the amounts of ROS and lipid peroxides is the key to their synergistic effect. First, the study discovered that PDT can promote lipid peroxidation in tumor cells and induce ferroptosis, and the degree of ferroptosis is correlated to the concentration of PUFA in cells [72]. Shui and his colleagues further studied lipid metabolism and found that the process of hypericin-PDT induced lipid peroxidation does not require the catalysis of classical ACLS4 and ALOXs [73]. Hypericin-PDT is still effective in tumor cells resistant to ferroptosis agonists. Ferroptosis caused by PDT is dependent on the concentration of photosensitizers. A low concentration mainly induces apoptosis, a high concentration induces necrosis, and an intermediate concentration mediates ferroptosis. Subsequent further studies on iron metabolism found that, unlike RSL3, the content of intracellular LIP showed a decreasing trend, but mitochondrial iron overload could be achieved through the mitochondrial membrane Ca^2+^/Fe^2+^ transporter (MCU). Since PDT treatment can down-regulate the expression of SLC7A11 and GPX4 to induce ferroptosis [74], and depletion of GSH can promote the effectiveness of PDT and ferroptosis [75], the System Xc^–^/GSH/GPX4 axis may be essential in PDT-induced ferroptosis.

More and more research has found that genes that regulate PDT can also control ferroptosis. The perspective of genes can not only provide direction for the discovery of novel ferroptosis regulatory genes but can also provide a new theoretical basis for enhancing the effectiveness of PDT. A study obtained four genes associated with ferroptosis (SLC2A1, SLC2A6, SLC7A5, ZEB1) by analyzing RNA-seq data after PDT in cholangiocarcinoma [76]. PDT can up-regulate the expression of SLC2A1, SLC2A6, and SLC7A5 and inhibit the expression of ZEB1, and four ferroptosis-sensitive genes are associated with the prognosis of patients with cholangiocarcinoma. Many previous studies have confirmed the negative role of histone deacetylases (HDAC) in PDT and ferroptosis. The use of HDAC inhibitory drugs can inhibit tumor recurrence and metastasis and induce ferroptosis [77,78]. The photosensitizer QpyNHOH, designed based on the inhibition of HDAC, can directly induce ferroptosis and increase the ROS production rate while simultaneously reversing intratumoral hypoxia and tumor antioxidant capacity [79]. Conversely, although knockdown of the expression of Yes-associated protein inhibited ferroptosis, it promoted tumor cell apoptosis and enhanced sensitivity to MPPa-PDT [80]. Combined it with erastin can alleviate the inhibitory effect on ferroptosis while promoting the antitumor effect of PDT. Another interesting study found a novel photosensitizer (TPCI) that does not rely on the activation of ACSL4 to cause lipid peroxidation. TPCI-PDT can activate ALOX12 by inducing ROS and down-regulating SLC7A11. It promotes lipid peroxidation of PUFA to induce ferroptosis [81]. In addition to being applied to tumors, ALA-PDT can also cause cell membrane rupture and thus enhance the killing effect of antibiotics. Further mechanistic studies found that PDT leads to the overload of Fe^2+^ in *M. abscessus* by up-regulating the expression of heme oxygenase MAB_4773, and it exhibits an antibacterial effect through ferroptosis [82].

PDT and ferroptosis can exhibit a synergistic effect by influencing immune cells and improving hypoxia, so improving TME is a research direction to further enhance the synergistic effect (Figure 2). First of all, immunotherapy is becoming a more significant component in the treatment of tumors. If anticancer therapy itself can activate the immune response, it will achieve longer-lasting and extensive effects. Photosens-PDT can kill tumor cells by inducing ferroptosis, and dead cancer cells can release damage-associated molecular patterns (DAMPs) including calreticulin, HMGB1, and ATP to further induce ICD [83]. Co-culturing with BMDCs found that tumor cells after PDT could promote the maturation and activation of BMDCs and the production of IL-6. In another study, PDT was combined with immune checkpoint blockade therapy and it was discovered that the combined treatment induced ferroptosis through reducing the expression of SLC7A11 and GPX4 [84]. In animal experiments, it not only successfully inhibits the original tumor but also efficiently suppresses the contralateral metastatic tumor. Combination therapy can also induce ICD to promote immune response. It can promote T cell infiltration and enhance the ratio of CD4^+^/CD8^+^ T cells by increasing tertiary lymphoid structures (TLS). The hypoxic environment of TME is one of the barriers that limit the efficacy of PDT and ferroptosis, and supplementing O_2_ can effectively alleviate this situation. Lu and his colleagues created a novel photosensitizer, the osmium–peroxo complex (Os2), which can be converted into osmium complex (Os1) with antitumor effects under anoxic environments and release O_2_^•−^. Os2-PDT can simultaneously induce ferroptosis by photocatalyzing endogenous NADH, depleting GSH, and inhibiting GPX4 [85]. O_2_^•−^ is catalyzed by superoxide dismutase to generate H_2_O_2_ and O_2_. O_2_ can improve hypoxia, and H_2_O_2_ can participate in the Fenton reaction to further promote ferroptosis.

## 2. Nanoparticles Combining Ferroptosis with PDT

Although PDT is widely used in clinical practice, most of the currently used photosensitizers are porphyrin-based photosensitizers with aromatic conjugated structures, which have disadvantages such as low photostability, poor water solubility, low active oxygen production rate, and unsatisfactory tumor therapeutic effect [86]. In the past two decades, improving photosensitizers has been the focus of the PDT research field. The third-generation photosensitizers, represented by multifunctional nano-photosensitizers, exhibit good biocompatibility, cycle stability, tumor targeting, and ROS generation efficiency [87]. In addition, loading and targeted delivering of photosensitizers through nanomaterials combined with other therapeutic modalities is an ideal strategy to improve the efficacy of tumor therapy [88].

Similarly, in addition to the physical and chemical advantages of nanoparticles, ferroptosis nanoparticles can also improve the insensitivity or drug resistance of tumors to ferroptosis and reduce the side effects of ferroptosis on normal tissues, so they have good application prospects and potential for clinical transformation [89,90]. Moreover, ferroptosis nanoparticles can also be loaded with anti-cancer drugs or modified tumor selection molecules to exert drug combination therapy and tumor targeting effects [91]. In this section, we will discuss how nanomaterials combine PDT with ferroptosis to further amplify the advantages of both and make up for their respective shortcomings.

### 2.1. Nanoplatforms Loaded Sorafenib

Sorafenib is a multi-targeted oral multikinase inhibitor, which mainly acts on vascular endothelial growth factor receptor (VEGFR) and platelet-derived growth factor receptor (PDGFR) to inhibit angiogenesis, as well as blocking the B-RAF and RAF1 of the mitogen-activated protein kinase in tumor cells to inhibit tumor proliferation [92,93]. Recent studies have reported that it can act as a ferroptosis activator and thus play an antitumor role [94]. Sorafenib can inhibit the expression of SLC7A11 and GPX4, and it can induce ferroptosis by depleting GSH and excessively accumulating iron ions, ROS, and malondialdehyde (MDA) [95]. At present, combining it with photodynamic therapy is a hot research direction, including two aspects: (1) Combining photosensitizers with sorafenib through the construction of nano-platforms to achieve more sensitive drug release responses, more precise tumor targeting, and higher ROS production (See Table 1 for details). (2) Sorafenib is used for chemotherapy combined with photodynamic therapy to achieve better synergistic therapeutic effects.

Some studies have constructed a nano-platform loaded with sorafenib and confirmed that sorafenib can combine with PDT to exert a synergistic antitumor effect by inducing ferroptosis. Liu et al. used Fe^3+^ ion, tannic acid, and sorafenib to construct SRF@FeIIITA nanoparticles, and they confirmed that it can induce ferroptosis by reducing the Fe^3+^ to Fe^2+^, inhibiting the GPX4 enzyme, and generating LPO [96]. They adsorbed the photosensitizer methylene blue to this nanoparticle to generate SFT-MB, and they confirmed that a large amount of active oxygen was generated after 660 nm laser irradiation. In vivo experiments confirmed that it has greater biological safety, better tumor targeting, and suppression effects. Another study connected hemoglobin (Hb) with photosensitizer Ce6 and loaded sorafenib to construct a two-in-one nanoplatform (SRF@Hb-Ce6) [97]. The SRF@Hb-Ce6 nanoplatform induces ferroptosis by depleting GSH, producing LPO and MDA, and inhibiting GPX4. The in vivo experiments revealed that the SRF@Hb-Ce6 nanoplatform combined with PDT further reduced GSH and increased ROS and MDA levels in tumor tissue. This confirmed that PDT combined with ferroptosis has a combined antitumor effect. In addition, the study also explored the mechanism and found that PDT can increase the sensitivity of tumor cells to ferroptosis by increasing the secretion of IFN-γ and down-regulating the expression of transporters (SLC7A11 and SLC3A2) on the cell membrane. Liu et al. loaded Ce6 and SRF into a red-blood-cell-derived vehicle (RDV) via endocytosis to construct Ce6@SRF@RDV nanoparticles [98]. Since an RDV has the structural characteristics of a biofilm, Ce6@SRF@RDV can be better taken up by tumor cells. This study confirmed that RDV can increase the production of singlet oxygen generation by supplying oxygen, thereby enhancing the antitumor effect of PDT. RDV can also enhance ferroptosis by supplying Fe and simultaneously promoting SRF to consume more GSH and produce more LPO and MDA. After Ce6@SRF@RDV nanoparticles were irradiated with a 660 nm laser, both in vivo and in vitro experiments confirmed that they could generate more ROS, and the antitumor effect was better than PDT or SRF alone. Wang et al. used an azobenzene (Azo) linker to covalently link chlorin e6 conjugated bovine serum albumin (BSA-Ce6) and ferritin to prepare a hypoxia-responsive nanoreactor, BCFe@SRF, loaded with sorafenib [99]. The study first confirmed its good biocompatibility and high targeting ability to TfR1 overexpressed tumors through in vivo and in vitro experiments, and it has good antitumor effects after laser irradiation. The degradation of Azo can release ferritin and sorafenib under hypoxic environments. Fe^3+^ in ferritin can consume GSH, while sorafenib can further consume GSH, produce ROS, and inhibit the expression of GPX4 so the nanoparticles can induce ferroptosis. Subsequent studies also confirmed that the ROS level of the nanoparticles after laser irradiation was higher than PDT alone, indicating that PDT and ferroptosis can simultaneously promote oxidative stress in a tumor and have a synergistic antitumor effect. Wang et al. constructed a tumor-targeting and NIR-triggered multifunctional nanoplatform (MnO_2_-SOR-Ce6@PDA-PEG-FA, MSCPF) using the photothermal agent MnO_2_, the photosensitizer Ce6, and sorafenib [100]. Under dual-wavelength laser irradiation (660 nm and 808 nm), MSCPF can inhibit tumor cells through strong heating and ROS, confirming that photothermal therapy (PTT) combined with PDT has a better antitumor effect. Compared with sorafenib alone or PDT/PTT treatment, MSCPF can produce more ROS, free Fe^2+^, LPO, and MDA in in vitro studies. It has a better inhibitory effect on HCC cells by inducing ferroptosis. MSCPF can inhibit the expression of SLC7A11, GPX4, and P-gp, thereby enhancing the accumulation of sorafenib in tumor cells and improving the antitumor effect. In vivo experiments also confirmed the synergistic anti-HCC effect of sorafenib and PDT/PTT. As previously reported, ferroptosis-inducing nanoreactors (Au/Fe-GA/Sorafenib@PEG) can disrupt the redox homeostasis in tumor cells by suppressing the expression of P-gp and GPX4 after laser irradiation. It can induce ferroptosis and effectively reverse drug resistance in cells [101]. Although this study only explored the synergistic effect of PTT combined with sorafenib, previous studies confirmed the combined effect between PTT and PDT. Loading photosensitizers on AFG/SFB@PEG nanoreactors may further enhance their antitumor effects and the ability to reverse drug resistance. Deng et al. used chimeric peptide (Pal-K(PpIX)-R4) and sorafenib to construct a plasma-membrane-targeted photooxidant (SCCP). They discovered that it could reduce the GSH by inhibiting the expression of SLC7A11 and GPX4 and produce ROS and LPO to induce ferroptosis [102]. Subsequently, a cytotoxicity test and flow cytometry confirmed that SCCP could have an antitumor effect through the synergistic effect of PDT and ferroptosis.

In addition, some studies have explored the synergistic effect of sorafenib nanoparticles combined with PDT from multiple perspectives. Although ferroptosis has not been directly explored, both sorafenib and PDT can activate ferroptosis, and ferroptosis is the future direction of research on this type of nanoparticles. Sun et al. constructed nanoparticles loaded with ce6 and sorafenib (NP-sfb/ce6). ROS can rapidly degrade the nanoparticles and promote the release of sorafenib under the action of a laser [103]. In vivo and in vitro experiments confirmed that NP-sfb/ce6 combined with PDT significantly inhibited tumor growth by boosting tumor cell death. The immunological response was further investigated, and it was found that PDT combined with NP-sfb/ce6 significantly increased tumor-infiltrating CD3^+^ T cells and decreased the percentage of MDSC (an important cellular component of immunosuppressive TME), thus enhancing T cell-mediated antitumor effects and improving TME. In addition, the synergistic antitumor effect of sorafenib and PDT may be related to the reduction of tumor angiogenesis. Another SC nanoparticle loaded with sorafenib and ce6 can generate photothermal reactions and ROS by PTT and PDT simultaneously. When combined with the antitumor angiogenesis effect of sorafenib, this nanoparticle can exert a synergistic effect [104]. Zhou et al. used BSA-modified photosensitizer MHI148 and sorafenib to construct the nanoparticle BSA-MHI148@SRF, which has better tumor targeting and biosafety [105]. The nanoparticles are mainly absorbed by mitochondria, which can reverse the hypoxic environment of tumors and enhance the efficacy of PDT by inhibiting mitochondrial respiration, reducing tumor oxygen consumption, and inducing normalization of tumor blood vessels. BSA-MHI148@SRF combined with PDT can reverse the tumor immunosuppressive microenvironment through the following two points: (1) It can induce ROS and suppress the production of PD-L1, which improves T cell infiltration and induces ICD. (2) It can inhibit VEGF and encourage the normalization of tumor blood vessels. Similarly, SCM@BSA nanoparticles (loaded with sorafenib, Ce6 and MnO_2_) combined with PDT also exerted a synergistic effect by improving the immunosuppressive response, which was mainly exhibited by the activation of cytotoxic T cells, T cells infiltration, and dendritic cells maturation [106]. SRF/Ce6-loaded PEG-M-PPMT NPs can be taken up by MMP-2-positive tumor tissues and cleaved in an acidic and high-intracellular-ROS-level environment to release sorafenib [107]. In vivo experiments found that PDT significantly inhibited tumor growth through reducing tumor blood vessels, and about 29% of tumors were completely eradicated. Another study constructed SPFT nanoparticles with cores composed of sorafenib and ZnPc(PS)4, and shells composed of FeCl_3_ and TA [108]. In an acidic environment, Fe^3+^ can be reduced to Fe^2+^ by TA. Fe^2+^ can be oxidized to Fe^3+^ through the Fenton reaction and produce toxic •OH. At the same time, sorafenib and ZnPc(PS)4 released by the degradation of nanoparticles can inhibit tumor growth through chemotherapy and PDT, respectively. In vivo experiments found that, compared with the free drug alone, the synergistic antitumor effect of SPFT was more significant. The Fe^2+^ produced by the nanoparticles in an acidic environment is also a key factor in inducing ferroptosis. Further research on the nanoparticles may provide a new basis for ferroptosis combined with PDT.

**Table 1:** Characterization of nanoparticles loaded with sorafenib.

Investigators	Nanoplatform	Average Particle Size (nm) (±S.D.)	Zeta Potential (mV) (±S.D.)	PDI **	Photosensitizer	Synergistic Effect	Cumulative Release of Sorafenib %
Wang et al. [100]	MnO_2_-SOR-Ce6@PDA-PEG-FA	117.09 ± (5.38)	−14.16 ± (2.47)	--	Ce6	Ferroptosis	~60 (pH = 5.5)
Wang et al. [99]	BCFe@SRF **	102.6 ± (1.3)	−2.7 ± (0.6)	0.28	Ce6	Ferroptosis	more than 90 (with Na_2_S_2_O_4_)
Liu et al. [96]	SFT-MB **	220	~−20	0.189	Methylene blue	Ferroptosis	68 (pH = 4, 72 h)
Xu et al. [97]	SRF@Hb-Ce6	175	−14.43	--	Ce6	Ferroptosis	more than 60 (with MMP2)
Liu et al. [98]	Ce6@SRF@RDV **	~190	~−31	--	Ce6	Ferroptosis	~90 (660 nm light)
Sun et al. [103]	NP-sfb/ce6	151.8 ± (11.4)	−23.6 ± (3.4)	--	Ce6	Enhanced tumor immune response	60.9 (After 120 h of laser irradiation)
Zhou et al. [105]	BSA-MHI148@SRF nanoparticles	145 ± (10)	−13.5 ± (1.1)	--	BSA-MHI148	Hypoxia reversion; Reverse the immunosuppression microenvironment	~80 (pH = 5.6, 24 h)
He et al. [106]	SCM@BSA **	181 ± (7)	−19.6 ± (0.4)	0.19	Ce6	Improves the immunosuppressive effect	~30 (pH = 6.5 + GSH)
Guo et al. [109]	CMV/C-S **	100	--	--	Ce6	Sensitize immune response (CI = 0.42)	83.7 (After 120 h of laser irradiation)
Shu et al. [107]	SRF/Ce6-loaded PEG-M- PPMT NPs	135.1 ± (3.0)	−3.3 ± (0.1)	0.241 ± (0.06)	Ce6	Antitumor angiogenesis	74.3 (irradiation at 500 mW/cm^2^)
Wu et al. [110]	SINP **	70 (dry state) 100 (swelling state)	--	--	Indocyanine Green	Increasing intracellular ROS level; Antitumor angiogenesis	--
He et al. [111]	SILs **	143.4 ± (3.9)	−12.4 ± (2.3)	0.123 ± (0.008)	Indocyanine Green	Antitumor angiogenesis	--
Yao et al. [108]	SPFT **	80	−28.07 ± (0.55)	--	ZnPc(PS)_4_	Chemotherapeutic Reactions	--
Yu et al. [112]	ZnPc/SFB@BSA nanocapsule	91 ± (21)	--	--	ZnPc	Chemotherapeutic Reactions	90.1 (with trypsin after 6 days)
Hu et al. [113]	Sor@GR-COF-366	165.4 ± (2.9)	−9.5	--	Porphyrin	Chemotherapeutic Reactions (CI = 0.22/0.26)	83.2 ± 3.8 (pH = 5.6, 48 h)
Wei et al. [104]	SC NPs **	~152	~−31.99	--	Ce6	Chemotherapeutic Reactions	--

** PDI: Polymer dispersity index; SRF: Sorafenib; SFT-MB: SRF@Fe^III^TA-methylene blue; RDV: Red blood cell-derived vehicle; SCM: Sorafenib, Ce6, MnO_2_; CMV/C-S: Cell membrane vehicle/Ce6 and sorafenib; SINP: Sorafenib/indocyanine nanoparticles; SILs: Sorafenib-Indocyanine Green liposomes; SPFT: Sorafenib/ZnPc(PS)_4_@Fe^III^-TA nanoparticles; SC NPs: Sorafenib and ce6 nanoparticles.

Sorafenib is being used as the first-line drug for the treatment of liver cancer at present, and some studies have confirmed its synergistic anti-hepatic effect with PDT by constructing photosensitizer-loaded nanoparticles. ZnPc/SFB@BSA nanoparticles can significantly inhibit the growth of HCC in in vivo and in vitro models [112]. SRF/ICG nanoparticles can promote ROS production and inhibit tumor angiogenesis and the growth of HCC [110]. The Sor@GR-COF-366 nano-platform combined with PDT has a 98% tumor inhibition rate in an orthotopic mouse HCC model [113]. SF–ICG liposomes encapsulated with sorafenib and ICG in nanoliposomes can inhibit HCC through PDT, PTT, and anti-angiogenesis of sorafenib [111].

Due to the limitations of ethical review and the long clinical trial cycle, it will take a long time for the above-mentioned nanoparticles to be used in clinical practice. At the same time, studies have explored the antitumor effect of directly using sorafenib for chemotherapy combined with PDT. A study explored the therapeutic effect of PDT combined with angiostatic kinase inhibitors through the chicken chorioallantoic membrane (CAM) [114]. Angiogenesis was observed in the CAM 24 h after PDT with complete vessel repair in 48 h. However, local application of sorafenib after PDT completely inhibited angiogenesis in the PDT-treated area, suggesting a potential antitumor effect of sorafenib combined with PDT. Subsequent studies confirmed this point, and using sorafenib after PDT can reduce vessel density and modulates vascular morphology, thereby significantly enhancing the inhibitory effect of PDT on human ovarian cancer and colorectal cancer [115]. The combination index of PDT combined with sorafenib is 0.59, indicating that they have a synergistic antitumor effect. In liver cancer cell lines (Huh-7 and Hep3b) and HCC patient-derived orthotopic xenograft (PDoX) mouse models, PDT combined with sorafenib also showed good synergistic antitumor effect (CI value < 1), and there were no obvious side effects [116]. Another interesting study found that photodynamic therapy improved skin toxicity induced by sorafenib [117]. The above studies show that PDT combined with sorafenib has a good prospect for clinical application.

### 2.2. Iron-Based Nanoparticles

Over the past decade, iron-based nanoplatforms have shown considerable promise due to their promising biosafety, targeting, and antitumor properties. When combined with other small molecules (such as photosensitizers, fluorescent dyes, chemotherapeutic medicines, etc.), iron ions can be employed as therapeutic agents for the Fenton reaction, photothermal therapy, and magnetic thermotherapy (MTH) [118]. As mentioned above, Fe^3+^ in the human body can be absorbed by transferrin, released in an acidic environment, and then reduced to Fe^2+^. The LIP is an important form of Fe^2+^ storage in the body. The increase in Fe^2+^ in the LIP can trigger the Fenton reaction, promote the production of ROS and LPO, and induce ferroptosis [119]. In the acidic tumor microenvironment, the Fenton reaction can effectively decompose hydrogen peroxide into hydroxyl radicals (˙OH) in tumor cells, which kills tumor cells [120]. In addition, Fe^3+^ can be reduced to Fe^2+^ by using GSH and H_2_O_2_. This redox process can release O_2_ while consuming GSH and improve the efficacy of PDT [121]. In summary, due to the key role played by iron ions in cellular redox, they can affect both ferroptosis and PDT. Recently, many iron-based nanoparticles have shown the potential for synergistic ferroptosis and PDT. In these studies, iron ions were introduced into nanoparticles in the form of Fe^3+^, Fe^2+^, Fe_3_O_4_, hemin, etc. The specific characteristics can be seen in Table 2, and they play a synergistic role through different mechanisms.

Sun et al. invented a new type of photosensitizer, SR780, which is specifically inactivated after conjunction with Fe^3+^. SR780@Fe-PAE-GP (loaded with Fe^3+^ and SR780, modified with glypican-3 receptor-targeting peptide) is a kind of nanoparticle with an “off/on” function [122]. It released Fe^3+^ and activated SR780 in an acidic environment. Fe^3+^ undergoes a redox reaction to generate Fe^2+^, which induces ferroptosis through the Fenton reaction. When combined with PDT, it can aggravate the oxidative stress in the tumor and exert a synergistic antitumor effect. Meanwhile, the depletion of GSH and the inhibition of GPX4 expression sensitized tumor cells to PDT. Under the synergistic treatment of PDT and ferroptosis, the inhibition rate of the PDX-HCC model was as high as 98.92%. Similarly, the Fe^3+^@Au1Ag24@PbP nanoplatform synergizes ferroptosis and antitumor effects of PTT and PDT by introducing Fe^3+^ ions [125]. Another study showed that p53/Ce6@ZF-T nanoparticles can induce ferroptosis in tumor cells through the Fenton reaction, and TA can reduce Fe^3+^ to Fe^2+^ to maintain the continuous occurrence of the Fenton reaction [126]. Fe^2+^-induced ferroptosis and Ce6-mediated PDT can continuously generate a large amount of ROS to kill tumor cells synergistically. At the same time, up-regulation of p53 protein expression can inhibit the expression of SLC7A11 and GPX4. Promoting the production of LPO further enhances ferroptosis. By amplifying the oxidative stress induced by ferroptosis and PDT, this study demonstrates that combination therapy is highly feasible. By increasing intracellular ROS and LPO levels while reducing GSH levels, PAF nanoparticles (polysaccharide as a carrier, TAPP as a PS) that contain Fe^3+^ increased Fe^3+^-mediated ferroptosis when combined with PDT [123]. Fe_3_O_4_ is a widely used nano-magnetic material. After decomposing in acidic TME, Fe_3_O_4_-PLGA-Ce6 nanoparticles combined with PDT can inhibit the expression of SLC7A11 and GPX4 and promote the expression of ACSL4. The tumor inhibition rate of the 4T1 tumor-bearing model in vitro was as high as 92.4%, indicating that the nanoparticles can synergistically enhance the effects of ferroptosis mediated by iron ions and PDT mediated by Ce6 [131]. FePtMn-Ce6/FA nanoparticles (FePtMn nanocrystals loaded with Ce6 and folic acid) can release Fe^2+^ in tumors. They can reduce H_2_O_2_ to •OH and produce LPO to kill tumor cells. They can also catalyze H_2_O_2_ to O_2_ to improve the hypoxic environment in the tumor [132]. Combined with Ce6-PDT, O_2_ can be catalyzed into ^1^O_2_ and then play a synergistic antitumor role. The above studies suggest that ferroptosis mediated by iron ions can enhance and sensitize the antitumor efficacy of PDT, which is an effective and widely used nano-therapeutic strategy. 

Hemin is an iron-containing porphyrin compound that can also be degraded to release iron ions. Hemin combines with G-quadruplex to form DNase, which can efficiently decompose H_2_O_2_ and generate O_2_ [142]. Xiao et al. designed a new DNA nanozyme (loaded with AS1411 nucleic acid aptamer and Ce6) based on the above reaction. Because AS1411 can specifically bind to nucleolin on the surface of tumor cells, it demonstrates effective tumor targeting [141]. Nanozymes can catalyze the decomposition of intracellular H_2_O_2_ into O_2_ to alleviate hypoxia and enhance the efficacy of PDT. They can also exert a synergistic antitumor effect by consuming GSH and inducing ferroptosis. HCNP (containing Ce6 and hemin) is a nanoparticle that can combine CDT, PDT, and ferroptosis. Hydroxyl free radicals (•OH) can be generated in the tumor microenvironment to generate CDT, which can activate ferroptosis with Ce6-PDT, which has good antitumor effects in vivo and in vitro [140]. 

However, the link between PDT and ferroptosis is not only shown after laser irradiation; some photosensitizers can also directly induce ferroptosis. Wu et al. found that the photosensitizer aloe-emodin (AE) can induce ferroptosis by inhibiting the activity of Glutathione S-transferase P1 (GSTP1) [135]. AE@RBC/Fe NCs were constructed by modifying red blood cell membranes and ferritin to AE, which could release ferritin and AE in an acidic environment and induce ferroptosis. After ferritin releases Fe^3+^, it can be reduced to Fe^2+^, which induces ferroptosis through the Fenton reaction. AE can reduce the activity of GSTP1, increase the amount of LPO, and damage mitochondria to induce ferroptosis. Subsequent PDT could further enhance AE@RBC/Fe NCs-induced ferroptosis by increasing intracellular ROS and consuming GSH. Another study also constructed Ferritin-Hijacking Nanoparticles (Ce6-PEG-HKN15) around ferritin. Although the nanoparticles do not wrap ferritin directly, the HKN15 peptide (ferritin-homing peptide) enables the nanoparticles to target and specifically gather around ferritin [137]. Subsequently, Ce6-PDT can degrade ferritin and activate ferroptosis, which cooperates with ROS to kill tumor cells. As in previous studies, the released iron interacts with excess intracellular H_2_O_2_ to generate O_2_, thereby enhancing photodynamic therapy and further amplifying the oxidative stress response.

Many previous studies have confirmed that ferroptosis is involved in many processes of antitumor immunity. Iron ions play an important role in immunity and can regulate the functional transformation of macrophages, neutrophils, NK cells, T cells, and B cells. The specific performance is to promote the conversion of macrophages to M1 type; promote the recruitment of neutrophils; maintain the function of NK cells; promote the differentiation of T cells; and promote the proliferation of B cells [143,144,145,146,147]. Therefore, the immune response can explain the synergistic effect between ferroptosis and PDT, which is a promising antitumor strategy. Novel nanomaterials (FeSe_2_–Ce6/MOF@HA/PEI/CpG@HHPA NPs) constructed from hydrogels exhibit good tumor aggregation and circulation stability due to negative surface charges [134]. When enriched in tumors, they can produce antitumor effects through the Fenton reaction and PDT. At the same time, combined with anti-CTLA-4 on the basis of CpG (immune adjuvant), they can also stimulate DCs to mature and activate CD4^+^ and CD8^+^ T cells. Antitumor immunity is exerted through the secretion of IL-6, IFN-γ, and TNF-α. A study discovered that Fe_3_O_4_@PGL NPs (iron oxide loaded porphyrin-grafted lipid nanoparticles) can be decomposed to Fe^2+^/Fe^3+^ in acidic lysosomes and then undergo a Fenton reaction with H_2_O_2_ generated by PDT [127]. Interestingly, when they co-cultured tumor cells with macrophages and added the nanoparticles, the production of ROS could be further increased and the antitumor effect was also stronger, so they believe that macrophages can significantly improve the antitumor effect of ferroptosis by accelerating the Fenton reaction in vitro. Fe_3_O_4_@Chl/Fe-CPBA CNPs (loaded with Fe_3_O_4_ and iron chlorophyll, modified with 4-carboxyphenylboronic acid) can deplete GSH and down-regulate the expression of GPX4 to induce ferroptosis and then kill bladder cancer cells by CDT [128]. Combined with PDT, they can also promote the production of lipid peroxides and enhance ferroptosis. The antitumor effect of this CDT–PDT therapy in in vivo experiments is excellent, and the survival rate of orthotopic MB49 tumor-bearing mice can be increased from 0 to 91.7%. Further studies found that CDT–PDT can inhibit PD-L1 to reverse the tumor immunosuppressive microenvironment. It reduced the recurrence of non-irradiated sites by inhibiting the expression of immunosuppressive factors, reducing the aggregation of M2-like macrophages, and inducing CD8^+^ T cells. Another study also found that Fe_3_O_4_ SAS@PLT nanoparticles can enhance the immune response by inducing ferroptosis, which has the potential prospect of synergistic application with PDT and PTT [129]. Similarly, PEG-encapsulated ferric hydride (PEG-Fns) nanoparticles can be triggered by blue light to release Fe^2+^ and induce ferroptosis, as well as activate the polarization of macrophages and induce the polarization of the tumor-promoting M2 type to the tumor-inhibiting M1 type [138].

Although nanoparticles can be aggregated with tumors through the EPR effect, they still have problems of low biological stability and poor targeting in clinical applications. Encapsulating nanoparticles with immune cell membranes can prolong blood circulation, enhance targeting, and reduce toxicity in vivo, which has become one of the directions to broaden the clinical application of nanoparticles in recent years [148]. He et al. constructed a novel biomimetic nanoplatform containing Ce6, hemin, and PEP20 (CD47 inhibitory peptide) and modified it with M1-type macrophage membranes. They found that Ce6-PDT can induce ferroptosis by promoting Fe^2+^ overload and inhibiting GPX4. Ferroptosis can be further enhanced under the stimulation of hemin [139]. This study found that a PEP20-mediated CD47-SIRPα blockade can stimulate IFN-γ secretion and inhibit System Xc^−^ to induce ferroptosis for the first time. In vivo experiments have confirmed that this can be combined with PDT to activate T cells and macrophages to exert antitumor immunity and effectively inhibit the growth and metastasis of primary tumors. Chen et al. used an 808 nm laser to decompose a photophage into three parts: (1) Fe_3_O_4_ was used as an iron source to activate a Fenton reaction to induce ferroptosis. (2) Black phosphorus nanosheet BPN, as a photosensitizer, can consume GSH through PDT and generate reactive oxygen species and lipid peroxides to enhance ferroptosis. (3) MnO_2_ can provide oxygen for PDT and relieve tumor hypoxia, thereby enhancing the antitumor effect [130]. Since the nanoparticles are also encapsulated by M1 macrophage membranes, they can increase tumor targeting and tumor cell uptake by prolonging the half-life of blood circulation and escaping from the immune system. Importantly, the results of in vivo experiments show that it can significantly inhibit tumor growth by promoting tumor-associated macrophage repolarization into M1 type, releasing TNF-α and IL-6 to initiate tumor immunity. In addition, cancer cell membranes can better camouflage nanoparticles to evade immune clearance and preferentially accumulate at tumor sites. Pan et al. used polyvinyl pyrrolidone, Fe^3+^, tetrakis (4-carboxyphenyl) porphyrin (TCPP), and tirapazamine to form PFTT. Then they wrapped particles with cancer cell membranes to form PFTT@CM nanoparticles. In TME, Fe^3+^ can react with GSH and H_2_O_2_ to generate Fe^2+^ and O_2_, respectively, which can enhance the effect of PDT by enhancing ferroptosis and improving the hypoxic environment [124]. Subsequently, further oxygen consumption by PDT can activate TPZ to induce DNA fragmentation, exhibiting a sequential synergistic therapy. Another study also used BPN as a photosensitizer, combined it with FePt nanoparticles, and modified it with polyethyleneimine (PEI) to form FePt/BP–PEI–FA nanoparticles [133]. Under 808nm and 660nm laser irradiation, it can exert the effects of CDT, PTT, and PDT. It exhibited a good synergistic antitumor effect through ferroptosis. In addition, the study also explored the combined effect of the nanoparticles and immunotherapy by combining with CTLA4 blockade therapy (CTLA4 can inhibit the ability of regulatory T cells). The PTT effect of FePt/BP–PEI–FA NC combined with CTLA4 blockade treatment could increase the percentage of CD4^+^ T cells and significantly activate the immune response, thereby effectively controlling the growth of primary and distant tumors (simulated metastatic tumor).

Elevation of ROS in the tumor microenvironment can induce tumor immunosuppression, which is an important conundrum limiting the application of PDT [149]. CPR nanoparticles (Ce6 as a photosensitizer, ferrocene as an iron agent, and RA as an antioxidant) have good tumor targeting and stability. These nanoparticles played the role of antioxidants to remove extracellular ROS before entering the lysosomes of tumor cells, and they enhanced the ICD induced by PDT and ferroptosis through activating HMGB1 [136]. On the contrary, in the acidic environment of tumor cell lysosomes, Fc induces ferroptosis through the Fenton reaction, and RA can reduce the Fc^+^ produced by ferroptosis to Fc in order to promote iron circulation. Combined with PDT, this can significantly increase the production of ROS and enhance the antitumor effect of PDT. Experiments in vitro and in vivo confirmed that CPR inhibited the EMT process of 4T1 tumor cells and reduced lung metastasis by inducing ICD. According to the research mentioned above, iron ions may be used as a connecting factor between ferroptosis, PDT, and immunotherapy, which has a positive synergistic antitumor effect.

### 2.3. Other Nanoparticles

In this section we will continue to discuss the application prospects of other types of nanoparticles combined with PDT and ferroptosis (See Table 3 for details). Recent studies have shown that not only can traditional Fe^2+^ induce ferroptosis through the Fenton reaction, but also that other metal particles (chromium, copper) can induce ferroptosis through the Fenton-like reaction [150,151]. Excessive glutathione concentration in tumor cells can scavenge ROS that exerts antitumor effects, and it is one of the important factors contributing to the poor efficacy of PDT [152]. The Fenton-like reaction can induce ferroptosis by consuming GSH, and the complexes formed by these metal particles also have the potential to be photosensitizers. Therefore, nanoparticles designed based on Fenton-like reactions may also exert synergistic antitumor effects with PDT. The coordination polymer IrS NP, constructed based on the IrIII complex, is biodegradable and tumor-accumulating. IrS NPs deplete GSH in tumors and inhibit GPX4 expression, which generates the LPO and induces ferroptosis in tumor cells. It was also found that the nanoparticles were mainly aggregated in mitochondria, and singlet oxygen and superoxide anion free radicals were generated after laser irradiation, leading to mitochondrial dysfunction and fracture. Therefore, IrS NPs can act as ROS scavengers, ferroptosis inducers, and photosensitizers to synergize PDT with ferroptosis [153]. Similarly, another study also confirmed that the Ir^III^ complex can induce ferroptosis through PDT. Yuan et al. constructed two novel benzothiophenylisoquinoline-derived cyclometalated Ir^III^ complexes (IrL1 and MitoIrL2), and Ir(III)-complex-mediated PDT under hypoxic conditions could induce ferroptosis by down-regulating GPX4, accumulating lipid peroxides, and shrinking mitochondria [154]. Surprisingly, Ir(III)-complex-PDT can suppress multi-organ metastatic tumors by enhancing immune responses. Ir-pbt-Bpa is a newly invented two-photon photodynamic photosensitizer which can activate ICD and exert antitumor effects [155]. By detecting ferroptosis-related indicators (ROS, GSH, LPO, GPX4), it was found that Ir-pbt-Bpa-mediated PDT killed human melanoma cells mainly through ferroptosis rather than apoptosis or necrosis. Further in vivo and in vitro experiments found that Ir-pbt-Bpa can increase the level of intracellular HMGB1/ATP, CD8^+^ T cells, and memory T cells, as well as reduce regulatory T cells in tumor-bearing mice, resulting in strong and long-lasting antitumor immunity.

Mitochondria, as the center of energy metabolism, is the subcellular structure that PDT and ferroptosis use to kill tumors. Tian et al. constructed HL/MOS@M780&LOD NPs (loaded with linoleic acid, hyaluronic acid, mesoporous silica, M780, and lactate oxidase) to target mitochondrial metabolism and inhibit tumor metastasis by consuming lactic acid and disrupting mitochondrial function [156]. Meanwhile, M780-PDT and LOD-mediated starvation can exacerbate tumor hypoxia, which, in turn, increases free iron ions by up-regulating TRF. Subsequently, GSH was consumed through the Fenton reaction to increase ROS, and linoleic acid was oxidized to LPO to induce ferroptosis. Enhancing ferroptosis by loading polyunsaturated fatty acids can enhance the antitumor properties of nanoparticles. Wang et al. used PLGA to wrap IR780 and modified human OS cell membranes to construct MH-PLGA-IR780 NPs, which can be better targeted and aggregated in tumors [157]. Compared with PDT alone, NPs combined with PDT can generate a large amount of ROS and damage mitochondrial function to exert a better antitumor effect. In addition, the PDT mediated by these nanoparticles can reduce the activity of GPX4 by inhibiting the System Xc^−^ transporter protein and activating NCOA4 mediated ferritinophagy to degrade ferritin, thereby promoting the accumulation of Fe^2+^ and inducing ferroptosis. The effect of ferroptosis is enhanced by directly interfering with crucial molecules in the process of ferroptosis. ML162 is a specific inhibitor of GPX4, and the nanomedicine C-ML162 constructed by combining it with Ce6 can accumulate at the tumor site and have strong therapeutic effects [158]. When irradiated with a laser, C-ML162 can produce a large amount of ROS and LPO. More importantly, ML162 can directly inactivate GPX4, weaken the cell defense system, and enhance ferroptosis. Another traditional GPX4 inhibitor, RSL3, was also used to construct nanoparticles to activate ferroptosis and induce immune response [159]. The nanoparticles can induce ferroptosis, promote ICD after laser irradiation, and enhance the sensitivity of tumor cells to ferroptosis by recruiting tumor-infiltrating T lymphocytes to secrete interferon-γ. In addition, combined with aPDL1, it can inhibit the stemness and invasiveness of tumor cells and effectively inhibit the growth of B16-F10 melanoma and the lung metastasis of 4T1 breast tumors. Inhibition of NRF2, which is also a key suppressor of ferroptosis, also enhances the antitumor effect of PDT. Brustol/silica@MnO_2_/Ce6@PDA-PEG-FA NPs can synergize PDT, PTT, and ferroptosis by improving hypoxia and inhibiting the oxidative stress defense system, and the tumor inhibition rate is as high as 86.7% [160]. MnO_2_ can provide O_2_ to enhance PDT to generate ROS, and it can also enhance PDA-PTT by inhibiting heat shock proteins. As an NRF2 inhibitor, Brustol inhibits GPX4 and FTH1, thereby promoting ferroptosis and enhancing PDT. Simultaneous inhibition of NRF2 can also inhibit HSP90 to enhance PTT. Therefore, nano-platforms that enhance ferroptosis by interfering with key molecules of ferroptosis (GPX4, ACSL4, System Xc^−^, NRF2, etc.) may be an important strategy for synergizing PDT and ferroptosis in the future.

A metal-organic framework (MOF) is a porous crystalline material with a three-dimensional structure, which uses metal ions as connection points and coordinates with organic ligands to form a porous crystal material. MOFs are ideal nanomaterials due to their topological diversity, high surface area, low density, ultrahigh porosity, and tunability [161]. Using disulfide-containing MOF as a nanocarrier to encapsulate Ce6 to construct Ce6@RMOF can deplete GSH through Ce6-PDT and thiol–disulfide exchange reactions in tumors. It can inhibit GPX4 and induce ferroptosis [162]. With the vigorous development of nanotechnology in recent years, nanoscale MOFs have become one of the research hotspots in the biological application of nanomaterials due to their good dispersibility, biocompatibility, biosafety, and bioactivity [163]. A series of porphyrin-based nano-organic frameworks, as a class of porous crystalline materials, have been found to have the potential to be photosensitizers, broadening the application prospects of NMOFs in PDT. Wang et al. used Buthionine-(S,R)-sulfoximine (BSO), porphyrin MOF, and HA to construct BSO-MOF-HA. MOF-PDT can generate ^1^O_2_, and BSO reduces GSH by inhibiting glutamate cysteine ligase. The combined effects can promote tumor cell oxidative damage and inhibit antioxidant defense, leading to disruption of tumor redox balance, thereby inducing ferroptosis [164]. Further studies found that ferroptosis and PDT can both mediate antitumor immunity by promoting CRT exposure and the secretion of HMGB1 and ATP. The combined therapy can also increase the proportion of mature DCs and activate cytotoxic T lymphocytes (CD4^+^ and CD8^+^ T cells). Porphyrinic MOF (PCN224) can be loaded with carbon-monoxide-releasing molecule 401 (CORM-401) and mitochondria-targeting amphiphilic copolymers and modified with hyaluronic acid to construct HA@MR@PCN-CORM [165]. PCN-224-PDT under laser irradiation generated a large amount of ROS and simultaneously triggered the rapid release of CO in the cells. CO can directly affect the content of GSH through glutamate cysteine ligase (GCL) and glutathione synthase, inducing ferroptosis and apoptosis. The tumor inhibition rate in in vivo experiments is as high as 90%.

Some nanoparticles also enhance the therapeutic effect on tumors by modifying or loading drugs. Phenylboronic acid (PBA) can selectively recognize and bind sialic acid that is highly expressed on the tumor surface. Au NRs/Cur/UCNPs@PBE will hydrolyze and release photothermal agent Au-NRs and photosensitizer curcumin in an acidic and ROS-rich TME. PTT and PDT can exert synergistic antitumor effects through ferroptosis under 808 nm laser irradiation [166]. Similarly, Au@Chl/Fe-CPBA nanorods (iron chlorophyll as a photosensitizer) also target glycoproteins on the surface of bladder cancer through CPBA and cooperate with CDT produced by the Fenton reaction to exert a synergistic antitumor effect after laser irradiation [167]. The nanoparticles are delivered by intravesical infusion, and PDT directly treats the tumor site, reducing systemic toxic reactions. Some nanoparticles enhance the antitumor effect by directly loading traditional chemotherapeutic drugs to induce chemotherapeutic responses at the tumor site. 5-Fu⊂nano DSPP-COF is decomposed by high levels of GSH in TME, releasing TFPP and 5-Fu to mediate PDT and chemotherapy responses, respectively. Synergistic therapy induces tumor cell ferroptosis by depleting GSH and inhibiting GPX4 expression [168]. GSH and thioredoxin (Trx) are two important antagonists of ferroptosis in cells under hypoxic conditions. C–N–Ce6 nanoparticle-mediated PDT produced ROS and induced ferroptosis, while NADPH-consuming micelles can consume GSH and Trx to destroy the redox homeostasis of tumor cells [169].

**Table 3:** Characterization of other nanoparticles.

Investigators	Nanoplatform	Average Particle Size (nm) (±S.D.)	Zeta Potential (mV) (±S.D.)	PDI	Photosensitizer
Ke et al. [153]	IrS NPs **	84 ± (4)	21.6 ± (0.9)	--	Ir^III^ complex
Zhong et al. [166]	Au NRs/Cur/UCNPs@PBE	114.13 ± (3.21)	12.67 ± (1.2)	0.342	Curcumin
Wang et al. [157]	MH-PLGA-IR780 NPs	236.8	−10.09 ± (0.70)	--	IR780
Zhao et al. [158]	C-ML162(ML162:Ce6 = 3:1)	140.1 ± (4.3)	~−25	0.223 ± (0.016)	Ce6
Yang et al. [165]	HA@MR@PCN-CORM	215	−20.8	0.085	PCN-224
Wang et al. [164]	BSO-MOF-HA	162.5	−25.5	--	MOF
Ding et al. [170]	MSP@ART@ P	~70	−13.6	0.061	ICG
Yu et al. [169]	C– N– Ce6 **	172.7 ± (4.9)	32.4 ± (1.0)	0.268 ± (0.011)	Ce6
Li et al. [168]	5-Fu⊂nano DSPP-COF	77	~ −15	0.181	TFPP
Song et al. [159]	BNP@R **	<100	--	<0.2	Pheophorbide a
Zhu et al. [9]	Ce6-erastin NPs	100 ± (20)	--	--	Ce6
Tao et al. [160]	BSMCPF **	--	--	0.207	Ce6
Meng et al. [162]	Ce6@RMOF **	--	--	--	Ce6
Tian et al. [156]	HL/MOS@M780&LOD NPs	--	−15	--	Mitochondria-targeted IR780
Liao et al. [167]	Au@Chl/Fe-CPBA nanorods	--	--	--	Iron chlorophyll

** IrS NPs: Ir^III^ complex nanoparticles; C– N– Ce6: p-nitrobenzyl chloroformate, chitosan, Ce6; BNP@R: PDPA and PDBA@ RSL-3; BSMCPF: brusatol/silica@MnO_2_/Ce6@PDA-PEG-FA; Ce6@RMOF: Ce6@glutathione-responsive metal organic framework.

## 3. Combined Strategies

### 3.1. Artemisinin

Artemisinin-based combination therapy is currently the first-line treatment for malaria. Since the advent of dihydroartemisinin (the most pharmacologically active artemisinin derivative), many studies have explored its potential therapeutic effect on tumors, inflammation, immunomodulatory diseases, and COVID-19 [171]. As a new type of ferroptosis agonist, it can induce ferroptosis in tumors by affecting cellular iron metabolism, promoting ROS production, and activating endoplasmic reticulum stress [172]. According to a study published in 2014, DHA can enhance the anti-esophageal cancer effect of PDT [173]. This study reported that DHA can inhibit NF-κB activated by PDT, thereby promoting the sensitivity of tumor cells to PDT. Through the detection of apoptosis indicators, it was found that the combination therapy significantly down-regulated Bcl-2 and increased the activation of caspase-3 and caspase-9, confirming that the combination therapy exerts a synergistic antitumor effect by inducing apoptosis. Based on the research on NF-κB, the team further explored the downstream mechanism of DHA for enhancing PDT. They found that PDT activates NF-κB and induces up-regulation of the expression of HIF-1α and VEGF, leading to tumor vascularization, thus weakening the therapeutic effect. The combination of PDT and DHA can reverse the antagonism of tumors to PDT and enhance the antitumor effect by inhibiting the NF-κB/HIF-1α/VEGF pathway [174]. The importance of this pathway was also confirmed in a study using 5-ALA-PDT combined with DHA to treat cervical cancer cells, and this study also discovered that 5-ALA-PDT combined with DHA can activate the NRF2/HO-1 pathway, which is different from our usual understanding that NRF2/OH-1 mediates tumor drug resistance [175]. Another study found that the combination of artemisinin and PDT can exert a synergistic antitumor effect by depolarizing the mitochondrial membrane and reducing the migration activity of tumor cells [176].

With further study on the pharmacological properties of artemisinin, it has been confirmed that it can induce ferroptosis and enhance the efficacy of PDT. Ding et al. loaded ART into porous magnetic supraparticles and modified the surface with dopamine, indocyanine green, and polyethylene glycol side chain to construct MSP@ART@P Nanodrug. After laser irradiation, it was found that the ROS level and antitumor activity could be significantly improved. This is mainly due to the endoperoxide bond (R-OO-R′) in ART and Fe^2+^ in MSP to generate ROS through the Fenton-like reaction [170]. Ce6-PDT can induce DNA damage response, and then up-regulate the expression of GPX4, leading to the resistance of tumor cells to PDT. When DHA is combined with PDT, the expression of GPX4 is significantly decreased, and the amount of ROS is higher than in monotherapy. Both in vivo and in vitro experiments have good synergistic antitumor effects, and the above results indicate that DHA-induced ferroptosis can improve tumor resistance to PDT [177]. Another study found that PDT combined with DHA may synergistically inhibit the invasion and migration of glioma cells by inhibiting the expression of NHE1 [178]. The results of in vivo experiments also confirmed that PDT combined with DHA can not only inhibit the growth of primary tumors but also inhibit recurrent tumors. Interestingly, they found that this synergistic antitumor effect could be reversed by NAC (a ferroptosis inhibitor). A study using 5-ALA-PDT combined with ART in the treatment of breast cancer also reached a consistent conclusion that the combined treatment can significantly increase the amount of ROS and inhibit the activity of tumor cells [179]. Using NAC can reverse the killing effect on tumor cells, but it does not affect the level of apoptosis. This confirmed that there are other types of cell death forms involved in the combined antitumor process. Therefore, ferroptosis may be the theoretical basis for the synergistic antitumor effect of artemisinin combined with PDT.

### 3.2. Other Combined Strategies

Sulfasalazine is a common immunosuppressant, which is mainly used clinically to treat autoimmune diseases, especially rheumatoid arthritis and inflammatory bowel disease [180]. In recent years, due to its ability to inhibit GPX4 and System Xc^–^, it has been used as a new type of ferroptosis agonist to exert antitumor effect [181,182]. Previous studies have found that SASP can reduce the content of intracellular GSH and enhance the anti-esophageal cancer effect of VP-PDT [183]. In another study exploring PHP-PDT against cholangiocarcinoma, it was found that PHP-PDT could up-regulate the expression of xCT in cholangiocarcinoma cells. After combined SASP, it can play a synergistic effect by reducing the level of GSH and increasing the content of ROS [184]. Based on the above studies, we believe that SASP may cooperate with PDT to promote ferroptosis by reducing the expression of xCT, but more research is needed to explore the specific mechanism of the combined therapy.

Erastin is the most classical ferroptosis agonist and promotes lipid peroxidation by depleting GSH. Erastin can mediate ferroptosis by affecting the cystine–glutamate transport receptor, the voltage-dependent anion channel, and p53 [185]. In an experiment using HOS human osteosarcoma cells, it was found that PDT alone increased the expression of YAP without affecting ferroptosis-related proteins. The level of apoptosis induced by PDT increases after YAP silencing, but it is also accompanied with the up-regulation of GPX4 [80]. The killing effect on tumors was further improved after being combined with erastin, which indicated that ferroptosis induced by erastin and apoptosis induced by PDT were cumulative. Subsequent studies further explored the Combination Index of the combination of PDT and erastin and found that the CI values of both the Ce6/erastin mixture and the Ce6-erastin nanoparticles were lower than 1, indicating that the combined therapy has good synergistic antitumor effects [9]. Erastin combined with PDT can generate a large amount of ROS. Using exosomes to encapsulate erastin can further improve its tumor targeting and circulation stability. After using CD47 surface functionalization (ExosCD47) exosomes to encapsulate erastin and a photosensitizer (Rose Bengal), both in vivo and in vitro experiments showed that ferroptosis in tumor cells was significantly induced; therefore, using exosomes as a delivery platform for photosensitizers and ferroptosis agonists is a promising strategy [186].

Similar to artemisinin, oxidants with anti-cancer effects extracted from natural plants may also promote the efficacy of PDT. A study found that low-dose laser irradiation does not affect normal cells and tumor cells, but adding gallic acid after irradiation can kill tumor cells by inducing ferroptosis and has little effect on normal cells [187]. This is an ideal PDT synergistic strategy for epidermal tumors (breast cancer, melanoma), which can greatly reduce the side effects of PDT on normal cells.

## 4. Conclusions and Discussion

The vigorous development of ferroptosis in the last decade has attracted the attention of many scholars. PDT, a combination of a photosensitizer and a laser, is an important method of applying ferroptosis in practice. In this review, we summarized the relationship between it and some approaches to combined therapy. Firstly, PDT has the ability to induce ferroptosis in tumor cells, and further enhancement of ferroptosis can improve the antitumor effect of PDT. From the perspective of the mechanism, both ferroptosis and PDT rely on redox balance disorder in tumor cells to produce a killing effect, so genes such as Nrf2, KEAP1, HO-1, GPX4, and other genes can participate in the regulation of them simultaneously. Based on these genes, exploring the synergy between them is the correct research direction. Secondly, ROS and lipid peroxides are essential for killing tumors in both PDT and ferroptosis. The type I photochemical reaction caused by PDT produces H_2_O_2_, which can further promote the Fenton reaction and induce ferroptosis. After the combination therapy, the level of ROS and lipid peroxide can be increased to achieve a synergistic effect. Both PDT and ferroptosis can stimulate the activation of immune cells and modify the TME. Damaged tumor cells release DAMPs to enhance ICD as well as promote the maturation of mDCs and activate T cells to further enhance the immune response. For macrophages, combination therapy promoted M2-type polarization to M1-type. From a complementary perspective, conventional ferroptosis agonists are chemotherapeutic drugs, which inevitably have the disadvantages of poor tumor site enrichment rates and strong side effects. As a treatment for local tumor killing, the poor systemic effect of PDT also limits its application. Therefore, systemic chemotherapy combined with local PDT has significant therapeutic implications and potential applications.

The most extensively researched area of combined effects is the construction of nanotechnology-based delivery systems for photosensitizers and ferroptosis agonists. After loading photosensitizers, nanoparticles based on sorafenib, iron ions, MOF, etc. have demonstrated good synergistic antitumor effects in vivo and in vitro. On this basis, the synergistic antitumor effect of PDT and ferroptosis can be promoted by modifying the biofilm structure to enhance the tumor uptake rate, release oxygen through redox reactions to improve tumor hypoxia, and load immune adjuvants to further enhance the immune effect. Despite the rapid development of ferroptosis nanoparticles in the research direction and application field, there are still some problems that need to be solved urgently. The first is the safety issue that nanoparticles cannot avoid. Nanoparticles can target tumors through the EPR effect, but some important organs will still accumulate nanoparticles and cause toxicity. Fe_3_O_4_ nanoparticles significantly alter the normal morphology of mouse hepatocytes, increase fibrous tissue, and are potentially genotoxic [188]. Fe_2_O_3_ nanoparticles have also been reported to affect glycerophospholipid metabolism in rat livers [189]. Although pomegranate peel extract can alleviate liver toxicity, the liver toxicity of Fe nanoparticles is a problem that cannot be ignored [188]. In addition, the effects of Fe nanoparticles on blood pressure, lung function, kidney function, and spleen function still need more research to confirm [190,191,192,193]. Inflammatory response is also an indicator on which to evaluate the safety of nanoparticles. Fe_3_O_4_ nanoparticles can activate the complement system, leading to increased anaphylatoxins and increased inflammatory factors [194]. Fe_2_O_3_ nanoparticles increase macrophages, neutrophils, and lymphocytes and induce inflammatory responses in the lung [193]. Since many ferroptosis nanoparticles contain heavy metal elements, neurotoxicity of these particles must also be assessed. Studies have reported that Fe nanoparticles can inhibit the secretion of dopamine and the proliferation of nerve cells, as well as promote the expression of neuronal α-synuclein [195]. Fe_3_O_4_ nanoparticles can stimulate microglia to release cytokines [196]. In summary, it is imperative to develop new methods that can detect and predict the toxicity of ferroptosis nanoparticles. 

Nanoparticles of different sizes have different toxicity and therapeutic effects on tumors. Surprisingly, a study found that the inconsistency in the size of Fe_3_O_4_ nanoparticles affects its efficacy in vitro and in vivo. The ultrasmall (<~5 nm) nanoparticles can induce ferroptosis to kill tumor cells through higher levels of ROS and Fe^2+^ [197]. However, in vivo experiments found that 10 nm Fe_3_O_4_ nanoparticles had the best antitumor effect. The size of Fe nanoparticles can also affect their pharmacokinetics. Fe nanoparticles of different sizes were distributed differently in the kidney, liver, and spleen of rats [198]. The larger the size of the Fe nanoparticles, the more uric acid was produced and the lower the level of lactate dehydrogenase. It is important to explore the specific size of nanoparticles to meet the lowest toxicity and best therapeutic effect.

## 5. Future Perspectives

Both PDT and ferroptosis exert antitumor effects by regulating oxidative stress in tumor cells. We suspect that several PDT-regulating genes that have been confirmed may also regulate ferroptosis at the same time, such as Transglutaminase 2, Na/H exchanger 1, apoptosis-inducing factor [178,199]. The ferroptosis regulatory genes discovered are also expected to regulate PDT, but more research is needed based on these genes linking ferroptosis and photodynamics to broaden the mechanistic pathways and exert a stronger antitumor effect. 

The clinical transformation of nanomaterials is the most critical problem in tumor therapy. Although animal experiments have confirmed the biocompatibility and biosafety of these nanoparticles, their clinical application still requires rigorous ethical assessment and clinical trial investigation, which is a long process. Therefore, it is more promising to consider the use of currently clinically approved ferroptosis agonists combined with PDT. In addition to erastin, RSL3, sorafenib, sulfasalazine, and artesunin mentioned in this article, numerous novel chemotherapy drugs (cisplatin, neratinib, lapatinib) and conventional drugs (statins) have also been confirmed as ferroptosis agonist [200]. More research is needed in exploring their effects in combination with PDT. In addition, these drugs still have side effects. For example, sorafenib can cause impaired liver function, hypertension, diarrhea, and skin toxicity [201,202]. Sulfasalazine can cause gastrointestinal and central nervous system reactions, hematopoietic depression, and fibropneumonia [203,204]. It is difficult to predict whether these side effects will affect their therapeutic effect and the prospect of application in combination with PDT. Ferroptosis agonists recently extracted from natural products may be a hotspot in the future. These substances have existed in nature for a long time and are widely consumed by people. Baicalin, piperlongumine, and tanshinone have been confirmed to induce ferroptosis and produce antitumor effects [205,206,207]. Many photosensitizers (pheophorbide A, phycocyanin, curcumin, resveratrol) are also obtained from natural products. Curcumin and resveratrol also act as ferroptosis agonists to exert antitumor effects [208,209]. Natural products may be a good medium for combining ferroptosis with PDT. In conclusion, PDT and ferroptosis have synergistic antitumor effects and are a promising synergistic therapeutic strategy.

## Figures and Tables

**Table 2:** Characterization of iron-based nanoparticles.

Investigators	Nanoplatform	Average Particle Size (nm) (±S.D.)	Zeta Potential (mV) (±S.D.)	PDI	Photosensitizer	The Source of Iron
Sun et al. [122]	SR780@Fe-PAE-GP	132.4 ± (9.8)	~−25 (pH = 7.4)	--	SR780	Fe^3+^
Li et al. [123]	PAF **	135	−23.2	--	TAPP	Fe^3+^
Pan al. [124]	PFTT@CM **	201	−14.22	~0.2	TCCP	Fe^3+^
Chen et al. [125]	Fe^3+^@Au1Ag24@PbP NPs	130	−55.3	--	Au1Ag24	Fe^3+^
Yu et al. [126]	p53/Ce6@ZF-T	120.12 ± (10.75)	−5.67 ± (0.71)	--	Ce6	Fe^2+^
Liang et al. [127]	Fe_3_O_4_@PGL NPs	~10	--	0.191	Porphyrin	Fe_3_O_4_
Chin et al. [128]	Fe_3_O_4_@Chl/Fe CNPs	145.6 ± (31.3)	−44.5	--	Iron chlorophyll	Fe_3_O_4_
Jiang et al. [129]	Fe_3_O_4_-SAS@PLT	268.9 ± (8.9)	–22.1 ± (0.9)	~0.13	--	Fe_3_O_4_
Chen et al. [130]	Photophage	122.88	−30.13 ± (1.64)	--	Black phosphorus nanosheets	Fe_3_O_4_
Chen et al. [131]	Fe_3_O_4_-PLGA-Ce6 NPs	85	−30.1	--	Ce6	Fe_3_O_4_
Yang et al. [132]	FPMCF NPs **	7.8 ± (1.56)	–22.7	--	Ce6	FePtMn
Yao et al. [133]	FePt/BP–PEI–FA NCs	~140	~−10	--	Black phosphorus nanosheets	FePt
Zhang et al. [134]	FSMH **	100–120	−14.8	--	Ce6	FeSe_2_
Wu et al. [135]	AE@RBC/Fe NCs	112 ± (3)	−25.6 ± (2.5)	--	Aloe-emodin	Ferritin
Zhou et al. [136]	CPR **	79.9 ± (6.42)	−10.2 ± (2.4)	--	Ce6	Ferrocene
Zhu et al. [137]	Ce6-PEG-HKN15	84.1	−10.3 ± (0.86)	~0.2	Ce6	HKN15
Yang et al. [138]	PEG-Fns **	~20	--	--	--	Ferrihydrite
He et al. [139]	MP@CH/BSA NP	137.33	−10.17	0.278 ± 0.098 (PBS 24 h)	Ce6	Hemin, Up-regulation of HMOX1 expression
Chen et al. [140]	HCNPs **	122	−22.2 ± (1.8)	--	Ce6	Hemin
Xiao et al. [141]	CH/DF **	--	--	--	Ce6	Hemin

** PAF: PEGylated polygalacturonic acid, 5,10,15,20-tetrakis (4-aminophenyl) porphyrin, and Fe^3+^; PFTT@CM: Polyvinyl pyrrolidone, Fe-tetrakis (4-carboxyphenyl) porphyrin, tirapazamine, cancer cell membrane; FPMCF NPs: FePtMn-Ce6/FA nanoparticles; FSMH: FeSe_2_-Ce6/MOF@HA/PEI/CpG@HHPA nanoparticles; CPR: chlorin e6-conjugated β-cyclodextrin, ferrocene-terminated phenylboronic acid, rosmarinic acid-boronic acid; PEG-Fns: PEG-coated ferrihydrite nanoparticles; HCNPs: two porphyrin molecules, chlorin e6 and hemin; CH/DF: Ce6, Hemin/DNA nanoflower.

## Abbreviation

AA: arachidonic acid; Ada: adrenal acid; AE: aloe-emodin; BH4: tetrahydrobiopterin; CAM: chicken chorioallantoic membrane; CDT: chemodynamic therapy; DHODH: dihydroorotate dehydrogenase; DHFR: dihydrofolate reductase; DAMPs: damage-associated molecular patterns; FTH1: ferritin heavy chain 1; FPN1: ferroportin 1; FSP1: ferroptosis suppressor protein 1; FtMt: mitochondrial ferritin; GSH: glutathione; GPX4: glutathione peroxidase 4; GCH1: GTP cyclohydrolase-1; GSTP1: glutathione S-transferase P1; GCL: glutamate cysteine ligase; HO-1: heme oxygenase-1; HDAC: histone deacetylases; Hb: hemoglobin; LPO: lipid peroxides; ICD: immunogenic cell death; LIP: labile iron pool; MUFA: monounsaturated fatty acid; MDA: malondialdehyde; MTH: magnetic thermotherapy; MOF: metal-organic framework; NCOA4: nuclear receptor coactivator 4; PDT: photodynamic therapy; PTT: photothermal therapy; PTX: paclitaxel; PDGFR: platelet-derived growth factor receptor; PEI: polyethyleneimine; PBA: phenylboronic acid; RDV: red blood cell-derived vehicle; SLC7A11: solute carrier family 7 member 11; SLC3A2: solute carrier family 3 member 2; TLS: tertiary lymphoid structures; TME: tumor microenvironment; TCPP: tetrakis 4-carboxyphenyl porphyrin; VDAC: voltage-dependent anion channel; VEGFR: vascular endothelial growth factor receptor.
